# Possible causes of disparities in the risk and outcomes of COVID-19: Cytomegalovirus and aged immune phenotype

**Published:** 2020-08-29

**Authors:** Andrew P. Smith, Graham Pawelec

**Affiliations:** ^1^School of Psychology, Cardiff University, UK; ^2^Center for Medical Research, University of Tübingen, Germany

**Keywords:** COVID-19, Cytomegalovirus, Aged immune phenotype, Disparities, Risk

A recent report by Public Health England [1] describes disparities in the risks and outcomes of COVID-19 in England. The largest disparity found was for age (those over 80 years were ×70 more likely to die than those under 40). Deaths in care homes accounted for more than 25% of the COVID-19 deaths. In those of working age, males were twice as likely to die as females. Diagnosis rates and risk of death were also higher in deprived areas and in BAME groups. Death rates were high in healthcare workers and those who interacted with the public. Comorbidities (diabetes; CHD; kidney disease; pulmonary disease; and dementia) were common in those with COVID-19 on the death certificate.

Many risk factors reflect exposure rates and/or the impaired functioning of different organ systems. The aim of the present article is to propose another mechanism that can plausibly account for the above disparities. In all virus infections, immune system function will be a crucial factor. One must now ask whether there is a possible immunological mechanism that could account for the risk factors seen with COVID-19. A recent review [2] argues that the previous infection history may be a determinant of risk from COVID-19.

Human cytomegalovirus (CMV) is a highly prevalent beta-herpes virus that establishes life-long latent infections. Around 30% of young adults in developed countries are infected, increasing to >60% in the elderly. In LMICs, 100% of young adults are seropositive. CMV plays a significant role in “immunosenescence,” and it has been estimated that CMV infection accelerates attrition of the naive T cell pool, which is required for generating adaptive immune responses against a novel virus such as SARS-CoV-2, by approximately 20 years [2].

Mortality rates from Covid-19 have been shown to be increased in certain ethnic groups and differences in CMV seroprevalence may act as one potential factor in this regard. The rates of CMV seropositivity are very high in populations that have suffered high mortality rates from SARS-CoV-2 such as northern Italy, China, and Spain. The influence of CMV on the long-term health of females has been shown to be less significant than observed in males. CMV is also associated with immune senescence and has been linked to a range of cardiovascular and metabolic disorders that are risk factors for COVID-19. Moreover, in elderly nursing home residents, high CMV-reactive CD4+ T-cells were associated with an increased risk of respiratory viral infection, including other coronaviruses [3]. Epidemiological studies show a clear association between CMV serostatus and cardiovascular disease, which may be a mechanism that enhances the pathology of SARS-CoV-2 infection. Research has also shown interactions between CMV, hyperglycemia, and SARS-CoV-2 infection that could promote tissue damage and increase the risk of death in diabetics.

Another review addresses the relevance of psychological studies of the upper respiratory virus infections to the COVID-19 pandemic [4]. Psychological stress has been shown to increase susceptibility and symptom severity of the upper respiratory tract viral illnesses. There has been a sharp increase in mental health problems during the COVID-19 pandemic and it has also been shown that high CMV infection levels are associated with greater mental health problems. In many cases, the stress induced by preventing and managing COVID-19 will resemble PTSD, which has also been shown to lead to an aged immune phenotype [5].

A CMV vaccine is highly desirable to prevent its direct health effects. In the context of COVID-19, CMV could be routinely measured as an indicator of risk. Prevention and management of other factors (e.g., extreme psychological stress) which are risk factors for an aged immune phenotype are also major priority. In some studies, CMV has been shown to have a negative impact on vaccine responses in older people [6] and this should be taken into account when evaluating the efficacy of vaccines for COVID-19.

The issue of “immunosenescence” is more complicated and requires further investigation as it has often been considered the cause of increased incidence and severity of infectious diseases and poorer response to vaccination in older adults [7]. It is also likely to be relevant to increased solid cancers and autoimmunity with age. There have been many attempts to identify biomarkers of “immunosenescence” but the overall profile from many studies is that the associations between immune risk profiles and mortality are context dependent. However, a major clinical impact of specific aged immune phenotypes will be as biomarkers for identifying response to novel infections such as SARS-COV-2 [8], and the development of successful vaccinations [9].
